# Differential Regulation of Macropinocytosis by Abi1/Hssh3bp1 Isoforms

**DOI:** 10.1371/journal.pone.0010430

**Published:** 2010-05-10

**Authors:** Patrycja M. Dubielecka, Ping Cui, Xiaoling Xiong, Sajjad Hossain, Susanne Heck, Lyudmil Angelov, Leszek Kotula

**Affiliations:** 1 Laboratory of Cell Signaling, New York Blood Center, New York, New York, United States of America; 2 Flow Cytometry Core, New York Blood Center, New York, New York, United States of America; 3 Confocal Microscopy Laboratory, New York Blood Center, New York, New York, United States of America; New York State Institute for Basic Research, United States of America

## Abstract

**Background:**

Macropinocytosis, which is a constitutive cellular process of fluid and macromolecule uptake, is regulated by actin cytoskeleton rearrangements near the plasma membrane. Activation of Rac1, which is proposed to act upstream of the actin polymerization regulatory Wave 2 complex, has been found to correlate with enhanced macropinocytosis. One of the components of the Wave 2 complex is Abi1. Multiple, alternatively spliced isoforms of Abi1 are expressed in mammalian cells, but the functional significance of the various isoforms is unknown.

**Principal Findings:**

Here, using flow cytometric assay analysis for Alexa Fluor 647, we demonstrate that Abi1 isoforms 2 and 3 differentially regulate macropinocytosis. LNCaP cells expressing isoform 3 had increased macropinocytic uptake that correlated with enhanced cell spreading and higher Rac1 activation in comparison to cells expressing isoform 2. Isoform 2 expressing cells had decreased macropinocytic uptake, but demonstrated greater sensitivity to Rac1 activation. Moreover, more isoform 2 was localized within the cytoplasm in comparison to isoform 3, which was more associated with the plasma membrane. Activated Rac1 was found to specifically bind to a site in exon 10 of isoform 2 *in vitro*. Because of alternative mRNA splicing, exon 10 is absent from isoform 3, precluding similar binding of activated Rac1. Both isoforms, however, bound to inactive Rac1 through the same non-exon 10 site. Thus, Abi1 isoform 3-containing Wave 2 complex exhibited a differential binding to activated vs. inactive Rac1, whereas isoform 2-containing Wave 2 complex bound activated or inactive Rac1 comparably.

**Conclusion:**

Based on these observations, we postulate that Abi1 isoforms differentially regulate macropinocytosis as a consequence of their different relative affinities for activated Rac1 in Wave 2 complex. These findings also raise the possibility that isoform-specific roles occur in other Abi1 functions.

## Introduction

Macropinocytosis is a key cellular process responsible for extracellular fluid and macromolecule uptake [1], [2], [3]. Viruses, bacteria, and apoptotic fragments are internalized by macropinocytosis [4], [5], [6], [7]. Macropinocytosis is involved in numerous processes including nutrient uptake and degradation [8], down-regulation of plasma membrane receptors following ligand binding [9], and antigen processing and maturation of dendritic cells [5], [10]. In macrophage, epithelial, tumor, and other cell lines, macropinocytic uptake increases upon activation of growth factor receptors [11], [12], [13], [14], [15]. One important objective regarding the regulation of macropinocytosis is to determine the mechanism by which growth factor and mitogenic signaling is coordinated with actin cytoskeleton reorganization.

Involvement of the actin cytoskeleton in macropinocytosis is well established. Each step of macropinocytosis, from ruffle formation to engulfment of a ruffle into a macropinosome, is likely to be regulated by multiple actin polymerization/depolymerization events [16], [17], [18]. The Abl binding protein 1 (Abi1), also known as Hssh3bp1, [19], [20], [21] is a key component of several intrinsic complexes that regulate actin cytoskeletal remodeling near the plasma membrane [22]. Overexpression of Abi1 in NIH 3T3 cells inhibits macropinocytosis [23]. Numerous structurally distinct isoforms of Abi1 exist in mammalian cells [21], [24], [25] providing a potential diversity of Abi1-actin regulatory complexes, suggesting the possible existence of multiple mechanisms through which Abi1 might regulate macropinocytosis.

Abi1 participates in several multi-protein complexes that regulate the dynamics of actin polymerization [26]. Eps8-Sos1 [27], [28], NWasp [29], [30], and Wave complex [31], [32], [33] including both Wave 1 and Wave 2, have been proposed to form complexes with Abi1. Each of these actin regulatory complexes involves a different mechanism, although at least two of these mechanisms converge directly on Rac1 function. The first, Eps8-Sos1-Abi1 complex, exhibits Rac-specific guanine exchange factor (GEF) activity transducing signals from Ras to Rac and activating the latter [27]. The second, Abi1-containing Wave 2 complex, has been directly linked to the effect of Rac1 on increased actin polymerization at the plasma membrane at the site of control of lamellipodia formation [32], [33]. Mechanistically, it was hypothesized that activated Rac1 targets the Wave 2 complex to the plasma membrane where Rac1 is responsible for activation of Wave 2/Arp2/3-dependent actin polymerization [34]. Increased actin polymerization near the plasma membrane, which is associated with increased macropinocytic uptake, may explain the postulated role of Rac1 in regulation of macropinocytosis. In this regard it has been demonstrated that a dominant negative Rac1 (Rac1-T17N) downregulates fluid uptake, but constitutively active Rac1 (Rac1-Q61L) upregulates the process [35], [36]. In the Wave 2 complex, Rac1 does not interact directly with Wave 2, but binds to Rac1-binding proteins Sra-1 and Nap1, which form complexes with Abi1 [31]. Abi1 interacts with Wave 2 and couples Wave 2 complex to Abl kinase activity after cell stimulation, thus promoting Wave 2 phosphorylation [37]. Tyrosine phosphorylation may regulate Wave complex activity either by regulating conformation of the complex [37] or by regulating interactions among components of the complex such as Abi1 and Abl kinase [38] or Abi1 and the p85 regulatory subunit of PI-3 kinase [28].

We originally demonstrated expression of multiple isoforms of Abi1 [21], although the functional significance of the various isoforms has not been addressed. Here, we demonstrate that expression of isoform 2 and isoform 3 have opposing effects on macropinocytosis. Expression of isoform 2 and isoform 3 produced different effects on actin cytoskeleton dynamics resulting in changes in cell spreading activity, and in differences in cell morphology. We postulate that these differences are due to differential binding of activated Rac1 to Abi1 isoform-specific sequences, thus suggesting the possibility of differential regulation of Rac1 activation by the different Abi1 isoforms.

## Results

### Establishment of cell lines expressing different isoforms of Abi1

**Figure 1:**
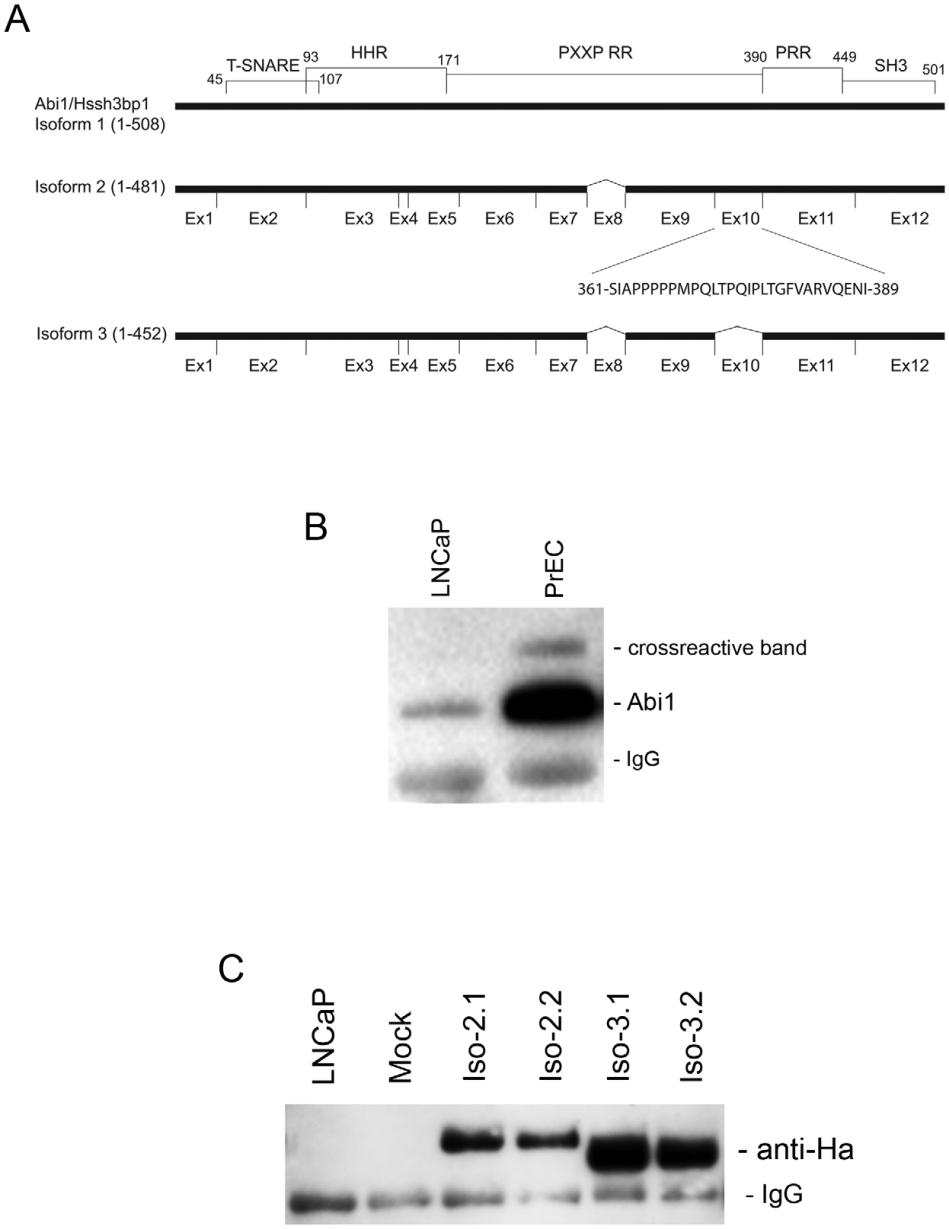
Expression of Abi1 isoforms in LNCaP cells. **A. Schematic comparison of Abi1 isoform 2 and isoform 3.** Comparison of the primary structure of isoform 2 and 3 indicates lack of exon 10 in isoform 3 (Ziemnicka-Kotula et al 1998). Exon 10 encodes a 29 amino acid proline-rich region. Abi1 contains several domains: T-SNARE, homeobox homology region (HHR), PXXP sequence-rich region (PXXP RR), Proline-rich region (PRR), and an SH3 domain. **B. Expression of endogenous Abi1 in LNCAP cells.** Abil was immunoprecipitated from lysates of the indicated cell lines with monoclonal antibody, 7B6, and blotted with polyclonal antibody, Ab-2. LNCaP indicates LNCAP cell line; PrEC indicates primary prostate cells; IgG indicates a cross-reactive IgG band. **C. Ectopic expression of Abi1 isoforms in LNCaP cells.** Ha-tagged recombinant Abi1 was immunoprecipitated from cell lysates with monoclonal anti-HA antibody and blotted with the polyclonal anti-HA antibody. Iso-2.1 and Iso-2.2, indicate independent cell lines expressing isoform 2 of Abi1; Iso-3.1 and Iso-3.2 indicate independent cell lines expressing isoform 3 of Abi1; Mock, indicates mock transfected cell line; LNCaP indicates the LNCAP cell line. IgG indicates a cross-reactive IgG band.

We previously defined expression of 5 isoforms of Hssh3bp1 [21], which was later referred to as Abi1. PCR based analysis and sequencing (not shown) revealed that several cultured cell lines such as Hela, A172, NIH 3T3, and the primary prostate cell line, PrEC, express mRNA species consistent with alternative splicing of Abi1 exon 10 involving isoforms 2, 3 and 5. Isoform 2 expressed exon 10 whereas isoforms 3 and 5 lacked exon 10 (Fig. 1A). Here we focused on elucidating the role of exon 10 because it represents a major structural difference between the Abi1 isoforms. The lack of isoform-specific antibodies precluded direct analysis of isoform-specific functions in tissues or cell lines. Therefore, we undertook a recombinant approach to analyze functional differences between isoform 2 and isoform 3.

To determine if functional differences among Abi1 isoforms existed, we generated LNCaP cell lines stably transfected with Abi1 isoform 2 or isoform 3. The LNCaP cell line exhibits very low endogenous levels of Abi1 expression. Endogenous expression levels of Abi1 in LNCaP cells are much lower than in primary prostate cells, PrEC (Fig. 1B). Thus we elected to transfect LNCaP cells with different Abi1 isoforms and to compare the function of these isoforms against a very low endogenous Abi1 background. Introduction of an HA tag at the C-terminus of recombinant Abi1 permitted screening of the clones as well as evaluation of relative levels of recombinant protein expression (Fig. 1C). In general, isoform 2 was expressed at lower levels than isoform 3 in stable transfectants.

### Macropinocytic uptake by transfected cells occurs in a time-dependent manner

**Figure 2:**
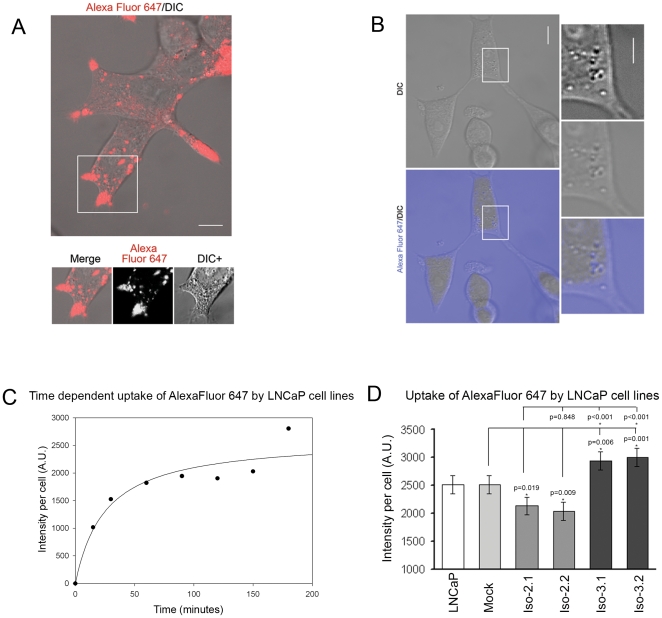
Uptake of Alexa Fluor 647 in LNCaP cells. **A. Live confocal observations of washed cells indicate that Alexa Fluor 647 accumulates in large vesicular structures enriched in cell extensions of LNCaP cells.** Representative merged image from DIC and Alexa Fluor 647 channel is presented. Boxed area is presented below. Merge, indicates merged image from DIC and Alexa Fluor 647 channel; Alexa Fluor 647, indicates Alexa Fluor 647 channel; DIC+, indicates DIC channel with enhanced contrast to better visualize vesicular-like structures. Bar, 10 µm. **B. Live confocal image of LNCaP cells immersed in cell culture medium containing Alexa Fluor 647.** Observations were carried out without removal of Alexa Fluor 647. Representative images from merged DIC and Alexa Fluor 647 channels (lower left panel) and DIC (upper left panel) are presented. Boxed area is presented as enlarged images in right panels: Lower right panel indicates merged image from DIC and Alexa Fluor 647 channels; middle right panel indicates DIC channel; upper right panel indicates DIC with enhanced contrast. Bar, 10 µm. **C. Flow cytometric analysis indicates time-dependent uptake of Alexa Fluor 647 in LNCaP cells.** Cellular accumulation of the fluorescent marker was determined by flow cytometry at the indicated time points following addition of Alexa Fluor 647 as detailed in Methods (n = 4, error bars ± s.e.m.). **D. Quantification of intracellular accumulation of Alexa Fluor 647 following a 2-hour incubation with the dye.** LNCaP, LNCaP cell line; Mock, mock transfected cell line; Iso-2.1 and Iso-2.2, isoform 2 expressing cell lines; Iso-3.1 and Iso-3.2, isoform 3 expressing cell lines. For each cell line measurements were performed on triplicate samples; error bars represent ± s.e.m.; Mock, n = 4; LNCaP, Iso-2.1, Iso-2.2, Iso-3.1, and Iso-3.2, n = 7.

In order to compare the role of Abi1 isoforms in macropinocytosis we applied both confocal microscopic and flow cytometric observations of living cells. Live observations were carried out following addition to the culture medium of a fluorescent, water-soluble dye (Alexa Fluor 647) as the macropinocytic target. Confocal imaging of cells obtained following washout of the labeled medium indicated apparent accumulation of fluorescence in large vesicular structures that were highly enriched in cell extensions (Fig. 2A). Live imaging of cells in the presence of Alexa Fluor 647 did not allow detailed analysis of cell extensions because of high background fluorescence. However, these observations indicated localization of fluorescent vesicles to DIC (differential interference contrast) (Nomarski)-negative structures in the cell body (Fig. 2B) (Supplementary Video S1 and Video S2). Distribution of the vesicular structures throughout the cell body and enrichment of fluorescent vesicles in cell extensions was confirmed by 3D reconstruction of labeled cells (Supplementary Fig. S1 and Supplementary Video S3). Interestingly, under the experimental conditions used no apparent diffusion of dye across the plasma membrane into the cell body, nor significant binding of the dye to plasma membranes was observed (Fig. 2A, 2B).

Time course experiments demonstrated the dynamics of macropinocytic uptake. Flow cytometric measurements obtained at different time points during incubation with the dye indicated that fluorescence reached steady state levels between 90–120 minutes of incubation (Fig. 2C). Macropinocytic uptake was inhibited by Latrunculin A treatment (data not shown), indicating that uptake was actin cytoskeleton-dependent [23].

### Abi1 isoform 2 and isoform 3 expression have opposing effects on macropinocytic uptake

Because transient high-level overexpression of Abi1 is known to inhibit macropinocytic uptake [23], we sought to determine if the different Abi1 isoforms differentially regulated macropinocytosis in LNCaP cell lines stably expressing Abi1 isoforms. As demonstrated in Figure 2D, uptake of the dye was significantly reduced in cells expressing Abi1 isoform 2, but was increased in cell lines expressing Abi1 isoform 3 in comparison to mock transfected cells or naïve cells. These data indicated that the two Abi1 isoforms under study had opposite effects on macropinocytic uptake: isoform 3 increased macropinocytosis, whereas isoform 2 reduced this process.

Control experiments using siRNA targeting of the recombinant Abi1 in isoform expressing cell lines restored macropinocytic uptake to the levels seen in mock or untransfected transfected cells. Targeting of total (both recombinant and endogenous) Abi1 by introducing siRNA specific to the coding sequence of Abi1 resulted in a significant decrease in macropinocytic uptake in all cell lines studied (not shown) indicating that Abi1 plays an important role in the process of macropinocytosis.

### The level of Rac1 activation correlates with macropinocytic uptake in Abi1 isoform-expressing cell lines

Rac1 and Abi1 are components of the Wave 2 complex that is proposed to regulate Arp2/3-dependent actin polymerization [31], [32]. The ability of Wave 2 complex to regulate actin polymerization is determined by the level of activated Rac1, which probably underlies the critical role of Rac1 in macropinocytosis [36], [39].

**Figure 3:**
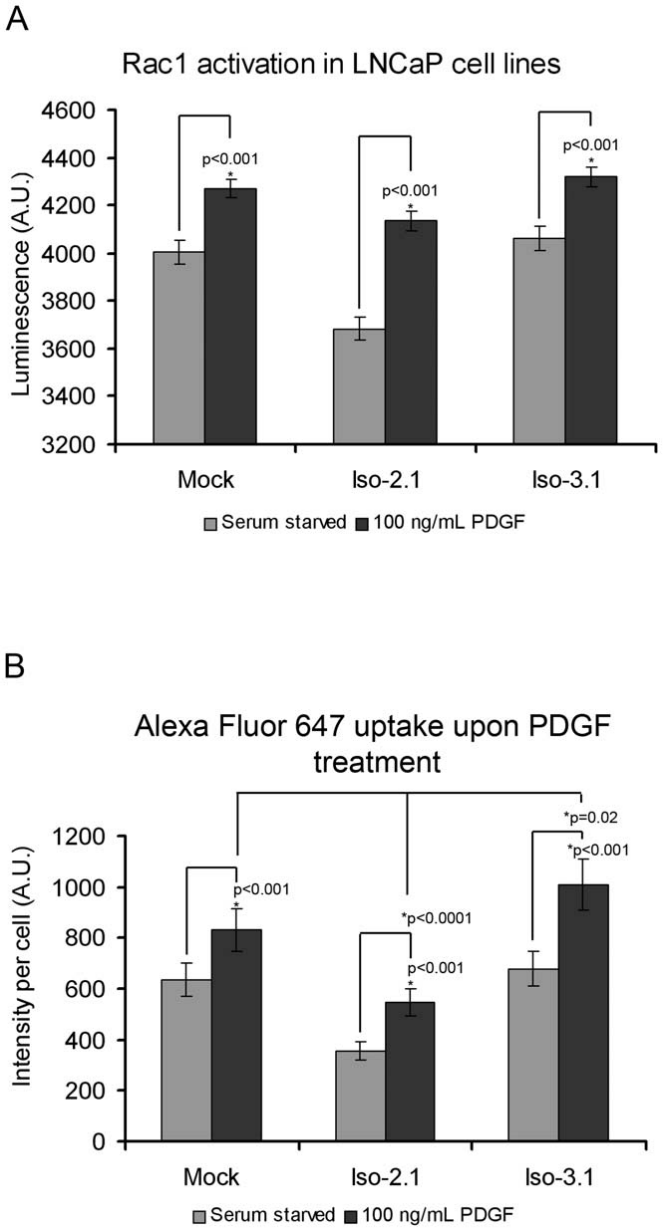
Abi1 isoform-specific regulation of Rac1 activation and interaction. **A. Isoform 2 expressing cell line exhibits lower levels of activated Rac1 but exhibits greater Rac1 activation in response to PDGF.** Serum starved and PDGF-induced Rac1 activity was evaluated by luminescence assay (Materials and Methods); PDGF was at 100 ng/ml. Measurements were performed in triplicate, error bars represent ± s.e.m., n = 4. **B. PDGF-induced Alexa Fluor 647 uptake is enhanced in Abi1 isoform expressing cell lines.** Cellular accumulation of Alexa Fluor 647 in LNCaP cell lines was evaluated by flow cytometry in serum starved or PDGF (100 ng/ml) treated cells following a 2-hour incubation with the dye. Mock, mock transfected cell line; Iso-2.1, isoform 2 expressing cell line; Iso-3.1, isoform 3 expressing cell line. Measurements were performed in triplicate, n = 3, error bars ± s.e.m.

To understand further the role of Rac1 and Abi1 in macropinocytosis, we examined levels of activated Rac1 in the Abi1-transfected cell lines. The line expressing isoform 2 had lower levels of activated Rac1 than did the line expressing isoform 3 or the mock transfected cell line. Although basal and maximal Rac1 activity was lower in isoform 2 expressing cells than in controls or isoform 3 expressing cells, platelet-derived growth factor (PDGF) induced greater Rac1 activation in isoform 2 expressing cells, as indicated by the fact that Rac1 activation was increased more over its basal level in isoform 2 expressing cells than in the other cell lines (Fig. 3A). To determine if Rac1 activation translated into physiological effects, we compared macropinocytic uptake in Abi1 isoform 2 and isoform 3 expressing cell lines following PDGF treatment. Consistently, macropinocytic uptake in Abi1 cell lines correlated with the Rac1 activation level, although the absolute level of uptake was a function of which Abi isoform was expressed (Fig. 3B).

### The activation status of Rac1 determines its interaction with Abi1 isoform-specific Wave 2 complexes

**Figure 4:**
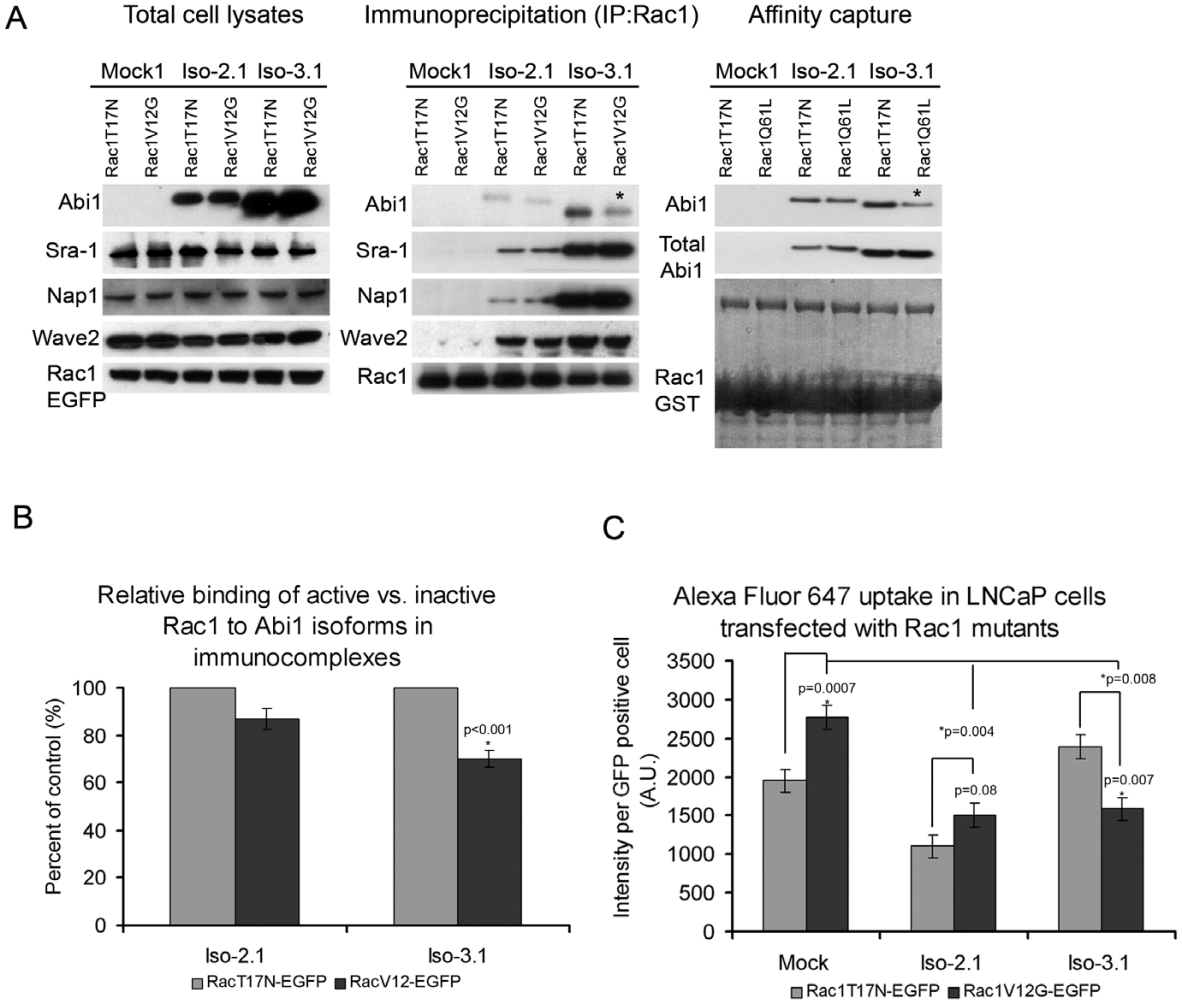
Active Rac1 interacts differentially with Abi1 isoforms. **A. Comparison of Rac1 mutant interactions with Abi1 isoforms.** Constitutively active Rac1-V12G (GFP-Rac1-V12G) or dominant negative Rac1-T17N (GFP-Rac1-T17N), were transfected separately and analyzed in Abi1 isoform expressing LNCaP cell lines as described in Materials and Methods. Membranes were blotted with anti-HA, anti-Sra-1, anti-Nap1 or anti-Wave2 antibodies to demonstrate the level of co-immunoprecipitated Wave 2 complex components; or with GFP antibody (GFP-Rac1-V12G, or GFP-Rac1-T17N) to demonstrate the level of immunoprecipitated Rac1. (Right panel) Immunoprecipitation results were further confirmed in affinity capture analyses where cell lysates from Iso-2.1 or Iso-3.1 expressing cell lines were subjected to pull down with either GST tagged Rac1 T17N (inactive) or Q61L (active) mutants used as baits. **B. Quantification of Rac1 binding to Abi1 isoforms in immunoprecipitation experiments.** Relative binding of active vs. inactive Rac1 to Abi1 isoforms was evaluated from proteins band intensities in three independent immunoprecipitation experiments. **C. Alexa Fluor 647 uptake in LNCaP cells transfected with active Rac1 shows opposite effects in Iso-2.1 compared to Iso-3.1 cell lines.** Measurements were performed in triplicate, n = 3, error bars ± s.e.m.

Rac1 activity is known to regulate Wave 2 complex activation [32], [34]. Activated Rac1 was previously shown to interact directly with two Wave complex components, Sra-1 [40] and Nap1 [41]. However, Abi1 is always present in the complex [32], [34] raising the question of whether the different Abi1 isoforms differentially interact with Rac1. To determine the role of the different Abi1 isoforms in the interaction of activated Rac1 with Wave 2 complex we transiently transfected Abi1 isoform expressing LNCaP cells with GFP-tagged dominant negative (T17N) or a constitutively active (V12G) Rac1 mutants. Immunoprecipitation using anti-GFP antibody revealed significantly reduced co-precipitation of active Rac1 vs. inactive Rac1 from cells that expressed Wave 2 complex containing Abi1 isoform 3 (Fig. 4A and 4B) suggesting that active Rac1 binds more weakly to, or dissociates more easily from, isoform 3-containing complex. No significant differences were observed in co-precipitation of active Rac1 or inactive Rac1 from cells that expressed Wave 2 complex containing isoform 2. Moreover, levels of Wave 2 complex components other than Abi1 were unchanged in cells expressing active vs. inactive Rac1 regardless of which Abi1 isoform was expressed (Fig. 4A). This suggested that the interaction of Rac1 with Abi1 isoforms in Wave 2 complex might depend on the activation status of Rac1.

To further confirm these observations we performed experiments in which total cell lysates from isoform 2 or isoform 3 expressing cell lines were subjected to pull down assays with GST tagged dominant negative (T17N) or constitutively active (Q61L) Rac1 mutants (Fig. 4A, right panel). Data obtained from these experiments were consistent with the immunoprecipitation data. Thus, both isoform 2 and isoform 3 bound Rac1, however, isoform 3 exhibited reduced binding of active Rac1 compared to inactive Rac1, whereas isoform 2 bound both forms comparably.

### Effect of Rac1 mutants on macropinocytic uptake in Abi1 expressing cell lines

We compared the effect of introduction of inactive and active Rac1 mutants on macropinocytic uptake in isoform 2 or isoform 3 expressing cell lines (Fig. 4C). In mock and isoform 2 expressing cell lines uptake was enhanced by co-expression of activated Rac1 compared to uptake in cells co-expressing inactive Rac1. In the isoform 3 expressing cell line, however, uptake was decreased in cells co-expressing activated Rac 1 compared to cells co-expressing inactive Rac1 (Fig. 4C). These results show that whereas co-expression of activated Rac1 increased macropinocytosis in control and isoform 2 expressing cells, co-expression of activated Rac1 reduced this process in isoform 3 expressing cells.

### Differential binding of active and inactive Rac1 to recombinant Abi1 isoforms

**Figure 5:**
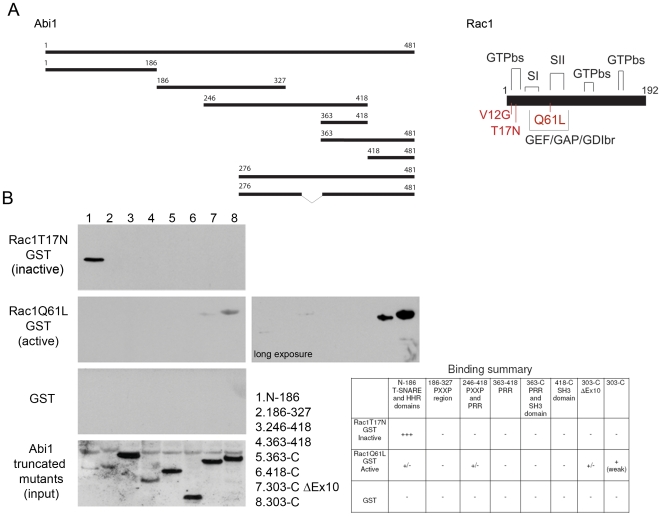
Mapping of Rac1 interactions with recombinant Abi1. **A. Schematic representation of Abi1 truncated mutants used for mapping and Rac1 structure.** A total of eight Abi1 constructs were used, each represents a major domain or region in the Abi1 protein (Fig. 1A). Rac1 contains three major GTP binding sites (GTPbs), two switch regions (SI and SII) and GEF/GAP/GDI binding region. Three mutations are depicted: N-terminal mutations T17N (dominant negative), V12G and Q61L (constitutively active). **B. Binding of Rac1 to Abi1 truncated products.** Inactive Rac1 binds to the N-terminal truncated mutant encompassing N-186 residues, and active Rac1 mutant shows exon 10 dependence in binding. Active Rac1Q61L binds weakly to this region. Active Rac1Q61L also shows exon 10 dependent binding to the 303-C region. Observations are summarized in Table below.

To gain mechanistic insight into functional differences between Abi1 isoforms we mapped their respective Rac1 binding sites using GST mutants of active and inactive Rac1 and recombinant fragments containing Abi1 domains (Fig. 5A). Inactive Rac1 mutant bound to the N-terminus of Abi1 (residues 1-186, Fig. 5B) whereas active Rac1 bound to the C-terminal Abi1 fragment containing exon 10 (residues 303-C-terminus, Fig. 5B). It is important to note here that binding of inactive Rac1 to the N-terminal Abi1 fragment was relatively strong despite its low solubility. On the other hand, binding of active Rac1 to the C-terminal fragment containing exon 10 was weak but consistently detected. This in vitro binding data provides an explanation for our finding that isoform 2 has higher affinity for active Rac1 than does isoform 3 (Fig. 4B). Thus, inactive Rac1 binds to the N-terminal domain of both Abi1 isoforms, but active Rac1 also binds isoform 2 through a site on exon 10.

### Differential cellular localization of Abi1 isoform 2 and isoform 3

**Figure 6:**
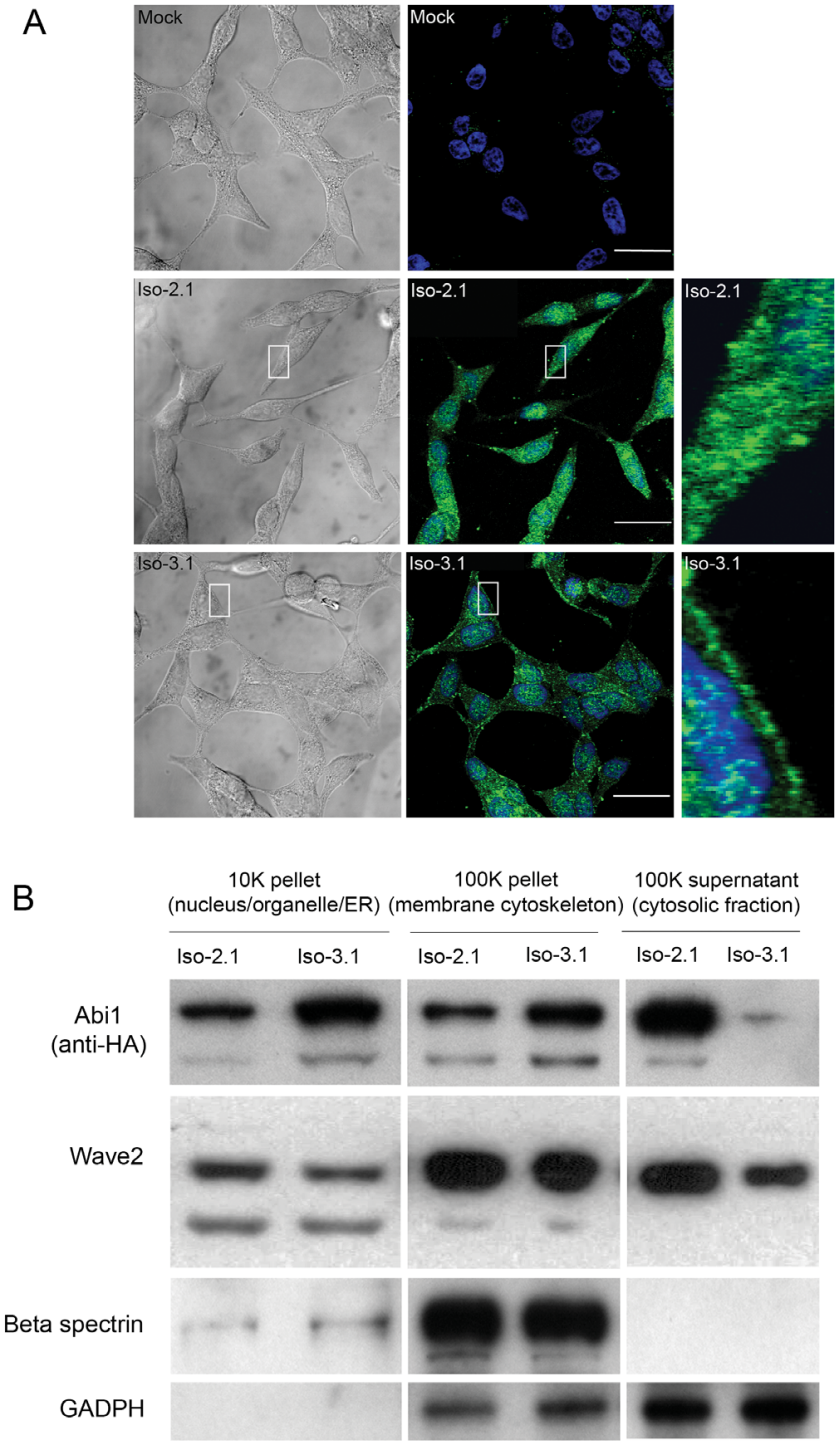
Differential subcellular localization of Abi1 isoforms. **A. Immunostaining.** Cell lines ectopically expressing recombinant isoform 2 and isoform 3 were immunostained with monoclonal antibody to HA (green). Representative images from DIC (left panels) and confocal channel (middle panels) are presented; boxed areas are enlarged on the right. Nuclei were stained with TO-Pro-3 (blue). Mock, mock transfected. **B. Subcellular fractionation.** Distribution of Abi1 isoforms in subcellular fractions obtained by indicated centrifugation steps (1 K = 1000×g, Materials and Methods). Analysis by Western blotting was performed using antibodies to: Ha (Abi1); Wave2, GAPDH (GAPDH); and beta spectrin (Spectrin). Transfected cell lines Iso-2.1 and Iso-3.1 were used for isoform 2 and isoform 3 subcellular fractionation, respectively.

Ha-tagging has permitted localization of recombinant Abi1 in stable cell lines with anti-HA antibody (Fig. 6A). A punctate staining pattern was observed for cells expressing either isoform. However, localization of the isoforms was significantly different: Abi1 isoform 2 showed enhanced cytoplasmic and perinuclear staining, whereas Abi1 isoform 3 demonstrated membrane and cytoplasmic staining, and appeared to have less concentrated staining in the perinuclear region than isoform 2 (Fig. 6A). Examination of Abi1 isoform distribution in LNCaP subcellular fractions confirmed the immunostaining results (Fig. 6B). Interestingly, Wave 2 showed slightly enhanced cytoplasmic localization in isoform 2 expressing cells compared to isoform 3 expressing cells. Thus, isoform 2 and isoform 3 show different subcellular localization.

### Isoform 2 and isoform 3 expressing cell lines exhibit different cellular phenotypes

**Figure 7:**
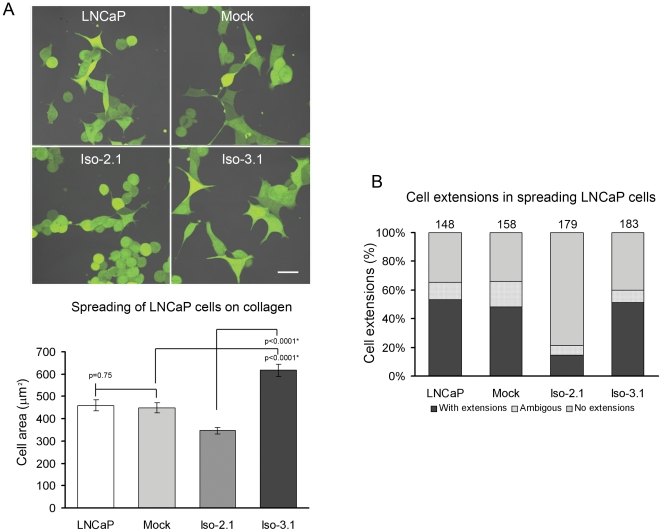
LNCaP cell lines expressing Abi1 isoforms exhibit different spreading and morphological phenotypes. **A. Isoform 2 expressing cell lines exhibit decreased spreading activity.** Cell lines were labeled with cell tracker and re-plated on collagen-coated dishes. Representative images from each cell line are shown. Mean cell area (n = 50, error bars ± s.e.m.) for each cell line was evaluated (graph) as described in Materials and Methods. Iso-2 cell line exhibited a significantly decreased mean cell area as compared to Iso-3 or mock control cell lines. **B. Isoform 2 expressing cell lines exhibit fewer numbers of cells with extensions during spreading.** The percentage of cells with large cell extensions/protrusions was evaluated in spreading cells (see Fig. 7A); round cells or cells with no discernable protrusion were scored as cells with no extension; overlapping cells were scored as ambiguous. Numbers of cells evaluated in each group are indicated above bars. LNCaP, naïve LNCaP cell line; Mock, mock transfected cell line; Iso-2.1, isoform 2 expressing cell line; Iso-3.1, isoform 3 expressing cell line.

Functional assays described in previous sections of this study indicated different actin dynamics in cell lines expressing different Abi1 isoforms. Strikingly, the cell lines also displayed significant phenotypic differences (Fig. 7). Based on our initial observations we evaluated these differences according to spreading activity and number of large cell extensions.

We noted that cells expressing Abi1 isoform 2 exhibit significantly reduced cell spreading activity. Upon replating of cells on collagen-coated culture dishes, cells expressing Abi1 isoform 2 were smaller than cells expressing Abi1 isoform 3 (Fig. 7A). It was also apparent that cells expressing Abi1 isoform 2 demonstrated decreased numbers of large cell extensions upon spreading in comparison to cells expressing Abi1 isoform 3. We quantified this by counting percentages of cells with and without extensions in each group (Fig. 7B). Interestingly, increased ability to form cell extensions during spreading activity correlated well with macropinocytic uptake.

## Discussion

Our results demonstrate that the differential cellular function of Abi1 isoforms in macropinocytosis can be explained by a distinct capability of association with active Rac1. We demonstrated that the isoform 2-specific interaction was dependent on exon 10, which defines the major structural and functional difference between Abi1 isoforms. Distribution of Abi1 isoform 2 was more cytosolic and perinuclear, and cells expressing isoform 2 showed reduced macropinocytosis and impaired cell spreading, whereas distribution of isoform 3 was at the cell membrane, and cells expressing isoform 3 exhibited greater cell spreading and increased macropinocytosis. These phenotypic differences can be explained by our mechanistic observations that indicate differential binding of active Rac1 to Abi1 isoforms thus suggesting the possibility of differential regulation of Rac1 activation in an Abi1 isoform-dependent manner.

**In our analysis, we have focused on observations of the endocytic vesicular compartment** [**23**] **rather than on membrane ruffling, as others have done** [36], [42], [43]. In addition, we did not use high molecular weight endocytic tracers such as fluorescently labeled dextrans or horse radish peroxidase [14], [15], or albumin [44], or molecules, such as wheat germ agglutinin that may nonspecifically bind to plasma membrane receptors, and thus activate intracellular signaling [45]. Instead, we used Alexa Fluor 647, which is relatively inert and is a non-plasma membrane permeable, water-soluble fluorescent dye. Application of live confocal microscopy as well as quantitative flow cytometric assays permitted analysis of macropinocytosis by evaluating uptake dynamics of Alexa Fluor 647 in LNCaP cells.

LNCaP cells appear to carry out macropinocytosis despite a mutation in the Abi1 gene [46] indicating that regulation of macropinocytosis is complex, probably involving multiple pathways. Although LNCaP cells exhibit a very low background expression of endogenous Abi1, Abi1 knockdown downregulates macropinocytosis in LNCaP cells (not shown) confirming that Abi1 is an important factor regulating macropinocytosis in LNCaP cells as was determined in other cell types [23], [29], [47]. In view of the low background of endogenous Abi1 expression, we reasoned that it was justified to compare directly the function of different Abi1 isoforms in the LNCaP cell line.

### Abi1 isoform-specific Rac1 binding affinities provide a basis for differential Rac1 activities

We demonstrated that a structural difference between isoform 2 and isoform 3—an alternatively spliced exon 10—underlies enhanced binding affinity of isoform 2 to activated Rac1. This might explain the increased Rac1 activation response to PDGF treatment in isoform 2 compared to isoform 3 expressing cells. It would also be consistent with enhanced accumulation of the dye in comparison to its basal state in the isoform 2 cell line. Cells expressing isoform 3 accumulated more dye and had higher initial Rac1 activities than did cells expressing isoform 2. This is consistent with the well-established hypothesis that activation of Rac1 promotes macropinocytosis [16], [35]. We postulate that the enhanced cytoplasmic localization of isoform 2 might lead to sequestration of active Rac1 in the cytoplasm, thus leading to lower levels of active Rac1 near plasma membrane and thus lower macropinocytosis.

### Association of Abi1 isoforms with Wave 2 complex provides a critical basis for the role of actin polymerization in the process

**Figure 8:**
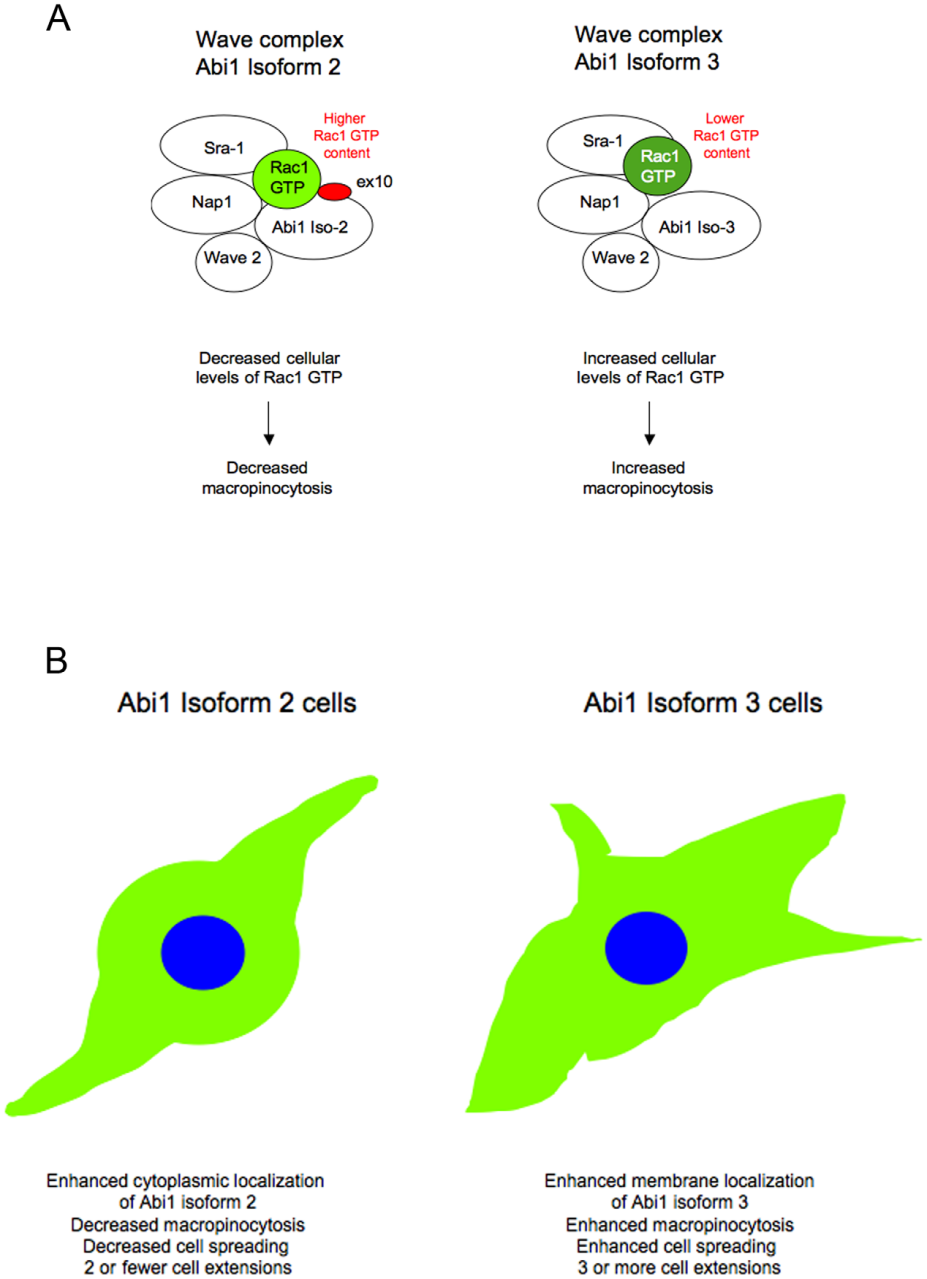
Model explaining differential function of Abi1 isoforms. **A. Abi1-Rac1 isoform-specific Wave 2 complexes.** Abi1 isoforms enter Wave 2 complex in an isoform-specific manner. Both isoform 2 and isoform 3 can enter Wave 2 complex, but isoform 2 provides an additional site in exon 10 (ex10) for interaction with active Rac1 (Rac1 GTP) as suggested by in vitro binding experiments. Active Rac1 was previously demonstrated to bind to two components of Wave complex, Nap1 and Sra-1. Isoform 2 sequesters active Rac1 in cytoplasm, thus leading to lower cellular levels of active Rac1 in isoform 2 expressing cells. **B. Phenotypic differences in Abi1 isoform-specific cell lines.** Cell lines expressing Abi1 isoform 2 or isoform 3 cell exhibit isoform-specific phenotypes.

Two other components of Wave 2 complex [31], [32], [34] were previously shown to interact with active Rac1, Sra-1 [40] and Nap1 [41]. Our results indicate that Abi1 might play an additional role in binding to active Rac1 in the complex (Fig. 8A). This hypothesis is strengthened by the fact that levels of other Wave 2 complex components in immunoprecipitated samples containing active vs. inactive Rac1 appear not to change but Abi1 isoforms do (Fig. 4A and 4B).

### Abi1 binds to inactive Rac1 thus suggesting its potential role as GEF in protein complexes

Both Abi1 isoforms share a binding site for inactive Rac1. Guanine nucleotide exchange factors (GEFs) sequester the dominant negative mutant of Rac1 thereby functioning as Rac1 activators [35], [48], [49]. Our binding data suggest that Abi1 might act as Rac1 GEF by interacting with the complex p85/Sos-1/Eps8 with an apparent GEF activity [50]. The fact that inactive Rac1 binds to the N-terminus of Abi1 suggest the possibility that Wave 2 competes with Rac1, since the region containing the Rac1 binding site includes the Wave 2 binding site [32].

### Abi1 isoforms enter Wave 2 complex suggesting the possibility of differential regulation of Wave 2 complex

Co-immunoprecipitation data indicate that Abi1 isoforms participate in Wave 2 complex. The observation that several cultured cell lines and tissues express multiple isoforms of Abi1 [21] point to the possibility that multiple Wave 2 complexes, i.e., associated with different Abi1 isoforms, might be functional in cells. The differential localization of recombinant Abi1 isoforms 2 and 3 in LNCaP cell lines further supports this hypothesis. Moreover, upon transfection of inactive and active Rac1 mutants we observed opposite effects on macropinocytic uptake in Abi1 isoform expressing cell lines, suggesting differential regulation of Wave 2 complex-dependent actin polymerization by Abi1 isoforms. We propose that differential localization of Abi1 isoforms, together with their differential binding affinities to active Rac1, underlie the mechanism of differential regulation of macropinocytic uptake. The more prominent plasma membrane localization of Abi1 isoform 3 is consistent with this isoform promoting macropinocytosis. The reduced ability of isoform 2 to promote macropinocytosis is consistent with the preferential cytoplasmic localization and tighter association of isoform 2 with activated Rac1 (Fig. 8).

**In conclusion, we have shown here that Abi1 isoforms 2 and 3 differentially regulate macropinocytosis in LNCaP cells via a mechanism involving differential regulation of Rac1 activity.** The presence of Abi1 in several intrinsic complexes that regulate actin cytoskeletal remodeling, and the existence of several Abi1 isoforms raises the possibility of isoform-specific roles in other Abi1-specific functions.

## Materials and Methods

### Cell line

LNCaP cells, clone FGC, CRL-1740 (ATCC, Rockville, MD) were grown in 10 cm culture dishes in RPMI 1640 with 10 mM Hepes, 1 mM sodium pyruvate, and 2 mM glutamax (all from Invitrogen, Carlsbad, CA) supplemented with 10% fetal bovine serum (FBS) (Hyclone, Logan, UT).

### Antibodies and reagents

Alexa Fluor 647 hydrazide, tris (triethylammonium) salt, Alexa Fluor 555 goat anti-rabbit IgG, Texas Red-X phalloidin, TOPRO3, Alexa Fluor 647 or Alexa Fluor 488 conjugated phalloidin, and Lipofectamine 2000 Transfection Reagent were from Invitrogen (Carlsbad, CA). Monoclonal anti-HA and anti-His6 were from Roche Diagnostic Corporation (clone BMG-His-1, cat #11922416001, Indianapolis, IN); Rabbit polyclonal anti-HA, mouse monoclonal anti-HA, PDGF BB (cat #3201), and anti-GADPH antibodies were from Sigma-Aldrich (St. Louis, MO). Antibodies to Rac1 and actin were from Cytoskeleton, Inc (Denver, CO). Horseradish peroxidase labeled goat anti-rabbit IgG, goat anti-mouse IgG, goat anti-rat IgG, and Protein G plus agarose were from Thermo Fisher Scientific (Worcester, MA). Antibody 1G9 to Abi1 was from MBL International Corporation (Woburn, MA). Antibodies to Abi1 were as described in [38] (monoclonal 7B6), or in [23] (polyclonal antibody Ab-2). Rabbit polyclonal anti-PDGFRB (platelet-derived growth factor receptor, beta) was from Cell Signaling Technology (Danvers, MA). Protease inhibitor cocktail set I and III, His•Bind® Columns, BugBuster, and imidazole were from EMD Chemicals Inc. (Gibbstown, NJ). Vectashield mounting medium was from Vector Laboratories, Inc. (Burlingame, CA). Gluthatione Sepharose 4B was from GE Healthcare, Piscataway, NJ.

### Expression plasmids and clone selection

Plasmids expressing HA-tagged isoform 2 (NM_001012750.1) or isoform 3 (NM_001012751.1) of Abi1 [21] were subcloned into pEGFP-N3 (Clontech, Mountain View, CA), and the GFP sequences were replaced with an HA tag followed with a stop codon at the C-terminus. An empty vector after GFP removal was used as the control (Mock). Cells were transfected with plasmids using Lipofectamine 2000 and selected using geneticin (0.6 mg/ml). All presented data are from representative clones Iso-2.1 and Iso-2.2 for isoform 2 of Abi1; Iso-3.1 and Iso-3.2 for Isoform 3 of Abi1; Mock for mock cell line, and LNCaP for naïve LNCaP cell line. For in vitro binding and pull down assays, purified His-tagged (at N-terminus) Abi1 isoforms were used. GST-fusions of the Rac1-T17N and Rac1-Q61L [51], [52], [53] plasmids were obtained from Theresia Stradal.

### Abi1 truncation mutants and recombinant Abi1 purification

His tagged Abi1 truncated mutants were prepared using Gateway directional TOPO cloning system using pDEST-17 (Invitrogen). Recombinant Abi1 was purified using recombinant proteins using His•Bind® Columns according to the manufacturer's instructions. BL21-A1 (Invitrogen) bacterial strain was used for protein expression. BugBuster 10x was used to lyse the bacteria. Lysate with soluble protein fractions was loaded onto the His•Bind Columns. The column with bound protein was washed in buffer containing 500 mM NaCl, 20 mM Tris-HCl, 5 mM imidazole, pH 7.9. For the next wash, buffer with 500 mM NaCl, 20 mM Tris-HCl, 60 mM imidazole, pH 7.9 was used. Finally, protein was eluted using buffer containing 500 mM NaCl, 20 mM Tris-HCl, 1 M imidazole, pH 7.9. Proteins were dialyzed against PBS, pH 7.4. The average yield was approximately 0.1 mg from 500 ml of bacterial culture.

### Co-purification and affinity capture binding assay

Bacterial suspensions for GST tagged proteins (baits) (BL21) or His tagged Abi1 mutants (BL21-AI) were combined in 1∶1 ratio, centrifuged, and lysed in PBST (phosphate-buffered saline, 0.1% Triton X100, pH 7.4) containing BugBuster (EMD) and protease inhibitor cocktail set I or III. Bacterial lysates with soluble protein fractions were incubated with Glutathione Sepharose 4B (GE Healthcare) After incubation sepharose beads were washed three times with PBST buffer containing protease inhibitors. Sepharose beads with bound protein complexes were reduced in SDS sample buffer and subjected to SDS PAGE. His tagged Abi1 mutants were detected using mouse monoclonal antibody anti-His6 (Roche). For pull downs we used baits: GST Rac1-T17N, or GST-Rac1Q61L bound to sepharose. Cell lines were starved for 24 hours followed by stimulation with 100 ng/ml PDGF and harvested in ice cold buffer (50 mM HEPES, pH 7.5, 1% Triton X-100, 50 mM NaCl, 5 mM EGTA, 50 mM sodium fluoride, 20 mM sodium pyrophosphate, 1 mM sodium vanadate, 2 mM PMSF). Protein concentration was determined using the Bradford assay, and samples were incubated overnight with respective baits. Next, sepharose or His resin beads were washed in the same buffer as above and subjected to SDS PAGE analysis.

### Flow cytometric uptake assay of Alexa Fluor 647

Cells were seeded into 12-well culture plates at 4×10^5^ cells per well. After 24 hours, cells were washed (3×) with warm PBS (phosphate-buffered saline, pH 7.4) (37°C). Subsequently, medium without FBS, containing 25 µM Alexa Fluor 647 was added. Alternatively, the same medium but with 100 ng/ml of PDGF was used for transient 5 minutes stimulation or with 10 ng/ml for 2 hours along with the dye. Cells were harvested (trypsin 0.25%, 1 minute) every 15 minutes from 0 to 3 hours (time course experiments) or at specific time point following addition of the dye to medium. Cells were washed (1×) with prewarmed (37°C) PBS before harvesting. Collected cells were subjected to cytofluorimetric analysis (BD FACS Canto, Becton Dickinson, San Jose, CA) in which the relative cellular content of Alexa Fluor 647 was measured by fluorescence intensity. Intensity of internalized Alexa Fluor 647 in live cells was obtained as a mean per cell value for 10^4^ cells in each measurement. In the transfection experiments where cells were transfected with GFP tagged proteins, a positive Alexa Fluor 647 signal was evaluated in GFP positive cells only. Each experiment was independently performed with the same batch of Alexa Fluor 647 at least three times. Nonlinear regression fitting in Fig. 1A was done by using SigmaPlot ver.11 (2010) (Systat Software Inc., San Jose, CA 95110 USA).

### Immunofluorescence microscopy

For microscopic observations cells were grown on coverslips and fixed with warm 3.7% paraformaldehyde for 10 minutes, permeabilized in 70% ethanol, and blocked in 2% BSA in PBS. Slides were incubated overnight with rabbit polyclonal anti-HA antibodies (Sigma-Aldrich, St. Louis, MO) followed by goat anti-rabbit IgG-Alexa Fluor 555. For F-actin staining, phalloidin-Alexa Fluor 647 was used. Nuclei were stained using TOPRO3.

### Rac1 activation assay

Cells were plated in 12-well plates at 2×10^5^ cells per well and cultured for 48 hours. Cells were then starved in serum-free media for more than 12 hours and then stimulated for 5 minutes with 100 ng/ml PDGF. Cells were washed with PBS and resuspended in ice-cold IP buffer. Lysates were subjected to G-LISA Rac1 activation assay (Cytoskeleton Inc. Denver, CO). See Supplementary Materials and Methods for details.

### Rac1 transfections, PDGF stimulation, and Western Blotting analysis

pEGFP-V12G Rac1 and pEGFP-T17N Rac1 plasmids (Addgene, Cambridge, MA) were used for transient transfection experiments. Alternatively, LNCaP cell lines were starved for 24 hours and stimulated with 100 ng/ml PDGF. Cells transfected with Rac1 plasmids or stimulated with PDGF were washed and collected in ice-cold PBS following by lysis in IP buffer. One milligram of total protein from each lysate was pre-cleared with 5 µg of rabbit IgG and used for immunoprecipitation with 5 µg of anti-GFP rabbit polyclonal antibody or 5 µg of anti-p85 monoclonal antibody, and 50 µl of protein G plus agarose. Cells were lysed 24 hours after transfection and subjected to immunoprecipitation and evaluation by Western blotting [23]. Immunoprecipitates were washed with IP buffer. Samples were prepared and analyzed by Western blotting as described [21] except that NuPAGE gels (Invitrogen, Carlsbad, CA) were used.

### Subcellular fractionation

Cell lysates obtained from cell lines Iso-2.1 and Iso-3.1 (PBS, pH 7.4 containing 2% Triton X-100 and protease inhibitors) were centrifuged at 10,000×g; the pellet was collected and supernatant was further centrifuged at 100,000×g [54]. Fractions were solubilized in SDS buffer and analyzed by Western blotting.

### Cell spreading assay

Cells were labeled with Cell Tracker Green CMFDA according to manufacturer's protocol (Invitrogen, Carlsbad, CA, cat#C2925). Observations were carried out following re-plating of cells onto collagen-coated 8-chamber culture slides (BD Biosciences, San Jose, CA) for 6 hrs using a Zeiss 510 META confocal microscope. To analyze cell spreading, the mean cell area (50 cells per sample) was determined using Adobe Photoshop version 7.0.

### Data analysis

The significance of the data was analyzed using Student's *t*-test, and differences between two means with P<0.05 were considered significant. Error bars represent the standard error of the mean (s.e.m.).

## Supporting Information

Figure S1 Representative images from 3D reconstruction of Alexa Fluor 647 positive compartment in LNCaP cells. Z-sections, from -60 degrees to +60 degrees in 3-degree steps were obtained from LNCaP cells as described in Materials and Methods. Left panel shows representative image from merged DIC and Alexa Fluor 647 channels; right panel shows DIC channel with enhanced contrast to visualize vesicular structure. Supplementary Video S3 represents 3D reconstruction of Z-sections. Note the distribution of Alexa Fluor 647-positive vesicular structures throughout cell body and enhanced staining in distal portion of cell extensions. Bar, 10 µm.
(2.58 MB TIF)

Video S1 Live observation of LNCaP cells immersed in cell culture medium containing 25 µM Alexa Fluor 647 (Fig. 2B). Cells were incubated for 1 hr and observation was carried out without removal of Alexa Fluor 647. The video shows merged DIC and Alexa Fluor 647 channel. Note the localization of Alexa Fluor 647 to DIC (Nomarski)-negative vesicular structures (Supplementary Video S2). Time lapsed is shown in seconds.
(0.33 MB MOV)

Video S2 Live observation of LNCaP cells immersed in cell culture medium containing 25 µM Alexa Fluor 647 (DIC only).
(0.41 MB MOV)

Video S3 3D reconstruction of Alexa Fluor 647 positive compartment in LNCaP cells was obtained from Z-sections. Note the distribution of Alexa Fluor 647-positive vesicular structures throughout cell body and enhanced staining in distal portion of cell extensions. Bar, 10 µm.
(1.78 MB MOV)
