# Supplementary figures and images for: Differential Regulation of Macropinocytosis by Abi1/Hssh3bp1 Isoforms
Source: PLoS One. 2010 May 10;5(5):e10430. doi: 10.1371/journal.pone.0010430 (PMC2866655; doi:10.1371/journal.pone.0010430)

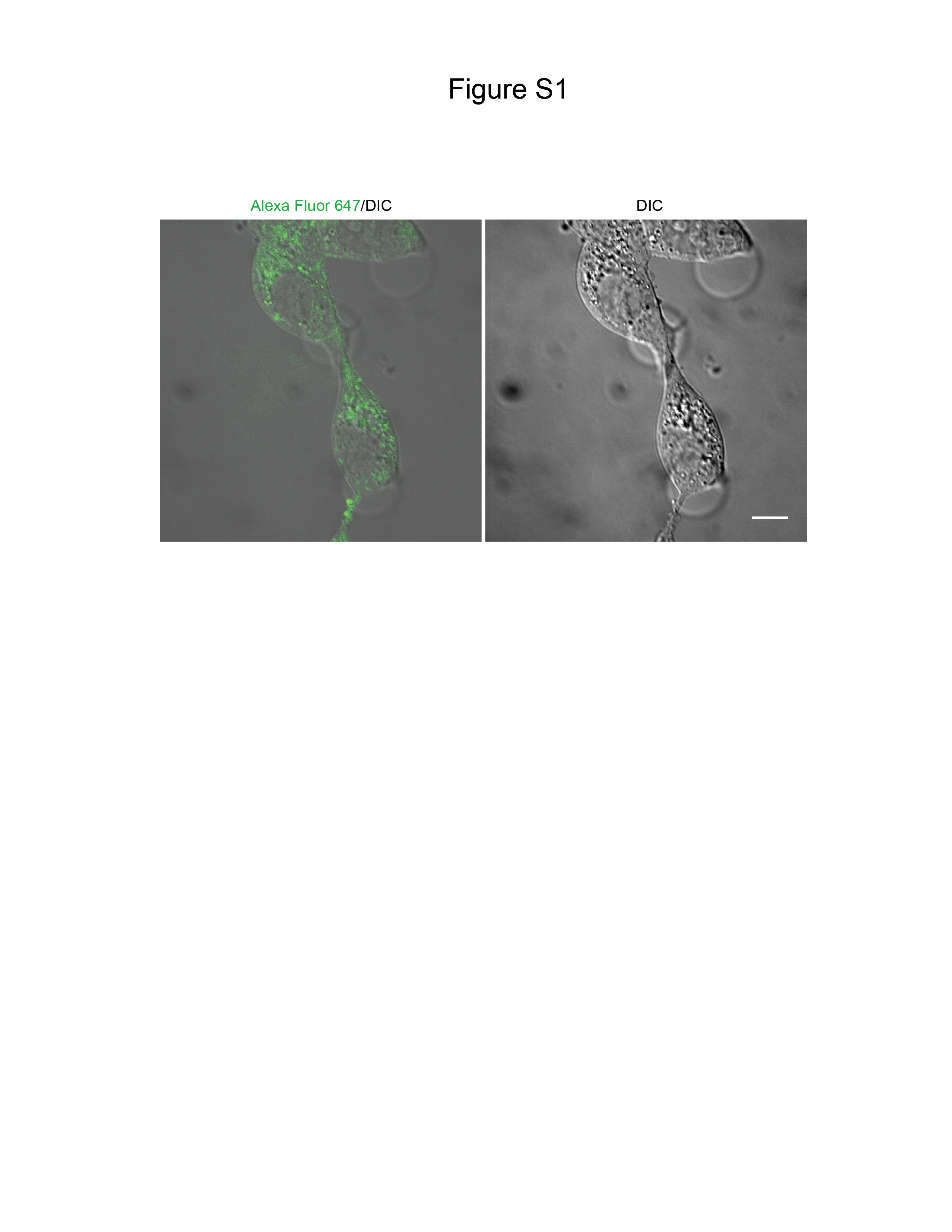

Supplement: Figure S1 — Representative images from 3D reconstruction of Alexa Fluor 647 positive compartment in LNCaP cells. Z-sections, from -60 degrees to +60 degrees in 3-degree steps were obtained from LNCaP cells as described in Materials and Methods. Left panel shows representative image from merged DIC and Alexa Fluor 647 channels; right panel shows DIC channel with enhanced contrast to visualize vesicular structure. Supplementary Video S3 represents 3D reconstruction of Z-sections. Note the distribution of Alexa Fluor 647-positive vesicular structures throughout cell body and enhanced staining in distal portion of cell extensions. Bar, 10 µm. (2.58 MB TIF) [file pone.0010430.s001.tif]
